# Well-founded practice or personal preference: a comparison of established techniques for measuring ulnar variance in healthy children and adolescents

**DOI:** 10.1007/s00330-019-06354-x

**Published:** 2019-08-07

**Authors:** Laura S. Kox, Sjoerd Jens, Kenny Lauf, Frank F. Smithuis, Rick R. van Rijn, Mario Maas

**Affiliations:** 1 Department of Radiology and Nuclear Medicine, Amsterdam UMC, University of Amsterdam, Meibergdreef 9, 1105 AZ Amsterdam, The Netherlands; 2 Academic Center for Evidence-based Sports medicine (ACES), Meibergdreef 9, 1105 AZ Amsterdam, The Netherlands; 3 Amsterdam Collaboration for Health and Safety in Sports (ACHSS), International Olympic Committee (IOC) Research Center AMC/VUmc, Meibergdreef 9, 1105 AZ Amsterdam, The Netherlands

**Keywords:** Adolescent, Child, Radius, Ulna, Radiography

## Abstract

**Objectives:**

Ulnar variance is a clinical measure used to determine the relative difference in length between the radius and ulna. We aimed to examine consistency in ulnar variance measurements and normative data in children and adolescents using the perpendicular and the Hafner methods.

**Methods:**

Two raters measured ulnar variance on hand radiographs of 350 healthy children. Participants’ mean calendar and skeletal ages were 12.3 ± 3.6 and 12.0 ± 3.7 years, 52% were female. Raters used the perpendicular method, an adapted version of the perpendicular method (in which the distal radial articular surface is defined as a sclerotic rim) and the Hafner method, being the distance between the most proximal points of the ulnar and radial metaphyses (PRPR) and the distance between the most distal points of both (DIDI). Intraclass correlation coefficients (ICCs) for intermethod consistency and inter- and intrarater agreement were calculated using a two-way ANOVA model. Variability and limits of agreement were determined using the Bland-Altman method.

**Results:**

The interrater ICC was 0.75 (95% CI, 0.61–0.84) for the adapted perpendicular method, 0.88 (95% CI, 0.80–0.93) for PRPR, and 0.94 (95% CI, 0.90–0.97) for DIDI. The intermethod consistency ICC was 0.60 (95% CI, 0.48–0.70) for perpendicular versus PRPR and 0.60 (95% CI, 0.49–0.70) for perpendicular versus DIDI. The intrarater ICC was 0.88 (95% CI, 0.70–0.95) for perpendicular, 0.90 (95% CI, 0.83–0.94) for PRPR, and 0.81 (95% CI, 0.69–0.89) for DIDI. The perpendicular method was not useable in 38 cases (skeletal age ≤ 9 years) and the Hafner method in 79 cases (skeletal age ≥ 12 years).

**Conclusions:**

The perpendicular and Hafner methods show moderate intermethod consistency. The Hafner method is preferred for children with skeletal ages < 14 years, with good to excellent inter- and intrarater agreement. The adapted perpendicular method is recommended for patients with skeletal ages ≥ 14 years.

**Key Points:**

*• The perpendicular method for measuring ulnar variance requires extended instructions to ensure good interrater agreement in pediatric and adolescent patients.*

*• The Hafner method is recommended for ulnar variance measurement in children with unfused growth plates and up to a skeletal age of 13 years, and the perpendicular method is recommended for children with fused growth plates and from skeletal age 14 and older.*

*• The mean ulnar variance measured in this study for each skeletal age group (range, 5–18 years) is provided, to serve as a reference for future ulnar variance measurements using both methods in clinical practice.*

**Electronic supplementary material:**

The online version of this article (10.1007/s00330-019-06354-x) contains supplementary material, which is available to authorized users.

## Introduction

Ulnar variance is a clinical measure that can be applied on hand radiographs to determine the relative difference in length between the radius and ulna. When the ulna’s relative length differs from that of the radius by less than 1 mm, this is termed neutral ulnar variance or “ulna zero” [1]. A deviation from this neutral position with the ulna exceeding the radius is termed positive ulnar variance, or “ulna plus” [2]. Consequently, a deviation in the opposite direction is termed negative ulnar variance, or “ulna minus.” However, exact values of ulnar variance and their interpretation depend highly on the method used to measure the ulnar variance. Population averages of ulnar variance vary around neutral and increase with grip [2–4]. Ulnar variance can be used to determine injury prognosis of distal forearm fractures [5] and in diagnosis of conditions like ulnar impaction syndrome and triangular fibrocartilage complex (TFCC) degeneration [6]. In young gymnasts with possible stress injury of the distal radius, ulnar variance is suggested to be on average more positive [7].

**Fig. 1:**
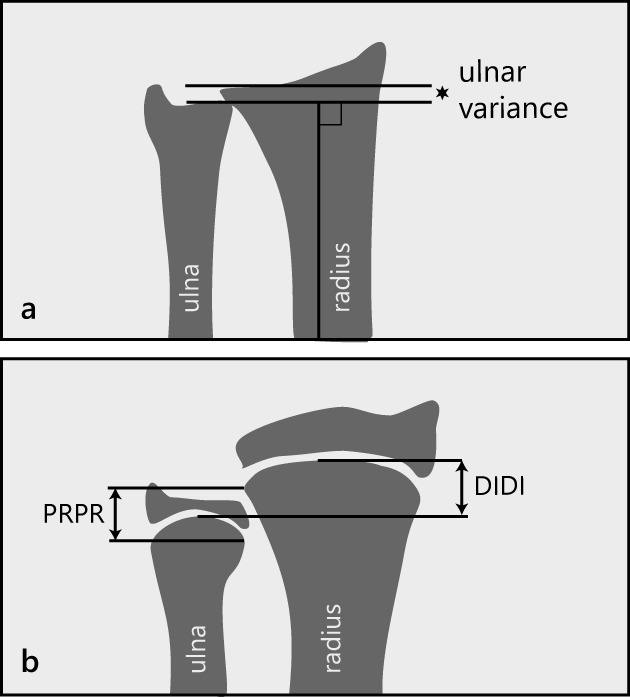
**a** The method of perpendiculars [11]. A line is drawn perpendicular to the longitudinal axis of the radius and through the most distal ulnar part of the radius. The position of the adjacent distal cortical rim of the ulna relative to this line is measured as positive, neutral, or negative ulnar variance. **b** The method of ulnar variance measurement as described by Hafner et al [12]. First, a line is drawn perpendicular to the longitudinal axis of the ulna and touching the most proximal point of the ulnar metaphysis. Similarly, a second line is drawn in the radius, perpendicular to its longitudinal axis and touching the most proximal point of the radial metaphysis. Ulnar variance is then defined as the distance between these two lines, in the literature often referred to as “PRPR” (“PRoximal-PRoximal,” distance “A”). Alternatively, the distance between the most distal point of the ulnar metaphysis and the most distal point of the radial metaphysis, often referred to as “DIDI” can be measured in a similar way (“DIstal-DIstal,” distance “B”)

Ulnar variance measurement methods include the “line technique” [8], the “concentric circle technique” [9], and the “method of perpendiculars” [10]. In the line technique, a line is drawn from the ulnar side of the articular surface of the radius to the ulna, and ulnar variance is defined as the distance between this line and the carpal surface of the ulna. The concentric circle technique uses a template of concentric circles placed with the center on the distal sclerotic line of the radius, and ulnar variance is measured by the distance between the line approximating the distal radius and the ulnar cortical rim. The perpendicular method measures the difference between two lines touching the distal ulnar aspect of the radius and the distal cortical rim of the ulna, both drawn perpendicular to the longitudinal axis of the radius. The latter was found to have the highest interrater and intrarater reliability of the three and is most often used in adults (Fig. 1a) [11]. However, in children, this method can be difficult to apply, because the distal radial and ulnar surfaces may not be (clearly) visible when the epiphysis is not fully ossified. To overcome this problem, a measurement method specifically for skeletally immature patients was developed by Hafner et al (Fig. 1b) [12]. This technique and the population data provided by this initial study have since been used in studies reporting ulnar variance in adolescent populations such as young gymnasts [7, 13].

The Hafner method has in turn been criticized for being unfamiliar to many clinicians, difficult to apply, and incomparable to values acquired in adult populations using other measurement techniques, while the perpendicular method showed good interrater reliability and was considered easy to apply in adolescents [14]. However, the perpendicular and Hafner methods have not been compared directly in pediatric or adolescent populations, nor have normative data been acquired from larger populations. For clinical use as well as for research on the possible relationship of positive ulnar variance and distal radial physeal stress injury, a reliable measurement method is essential.

As the only reference standard for ulnar variance is in vivo measurement of true ulnar and radial length, which is not favored or even possible in most cases, relative measurement suffices in daily clinical practice [9]. Such a measurement needs to be easily applicable and reliable, and to allow comparison with other populations measured with the same technique. This study aims to determine consistency of the perpendicular method for measuring ulnar variance and the Hafner method in a Western European pediatric and adolescent population and to provide normative population data for the distribution of measurements in children and adolescents for both methods.

## Materials and methods

### Design

This retrospective study included a random sample from a population of healthy children and adolescents of a previous study in which normal values for phalangeal radiographic absorptiometry were determined [15]. This study population consisted of children from the Erasmus Gymnasium in Rotterdam and children of employees (and their relations) at the Erasmus Medical Center Rotterdam. Inclusion criteria were inclusion in original study population by Van Rijn et al [15], and age 18 years or younger. Exclusion criteria were any disease or use of medication known to affect bone growth and/or metabolism (in accordance with the study by Van Rijn et al [15]), radiographically visible growth deformity of the wrist and/or hand, and radiographically visible upper extremity fracture. Ethical approval was obtained for the initial study and for subsequent use of the data. In keeping with national guidelines on clinical studies in children, informed consent was given by parents or guardians alone for children younger than 12 years, and by parents or guardians as well as the child for children aged 12 years or older. The included sample consisted of 185 girls (53%) and 165 boys (Table 1).

**Table 1:** Participant characteristics

	Total group (*n* = 350)	Girls (*n* = 185)	Boys (*n* = 165)
Calendar age in years (mean ± SD (range))	12.3 ± 3.6 (4.4–18.9)	12.6 ± 3.7 (5.2–18.9)	11.9 ± 3.4 (4.4–18.3)
Skeletal age in years (mean ± SD (range))	12.0 ± 3.7 (4.6–19.0)	12.5 ± 3.7 (5.0–18.0)	11.5 ± 3.7 (4.6–19.0)
Tanner stage (*n* (%))^a^			
1	117 (33%)	59 (32%)	58 (35%)
2	50 (14%)	20 (11%)	30 (18%)
3	35 (10%)	13 (7%)	22 (13%)
4	85 (24%)	49 (27%)	36 (22%)
5	63 (18%)	44 (24%)	19 (12%)
Hand dominance (*n* (%))			
Right	319 (91%)	171 (92%)	148 (90%)
Left	30 (9%)	13 (7%)	17 (10%)
Both	1 (0.3%)	1 (0.5%)	0

^a^Tanner stage: stage of physical development in children, adolescents, and adults based on external primary and secondary sex characteristics (e.g., size of the breasts, genitals, testicular volume, and development of pubic hair) [16, 17]

The primary outcome measure was intermethod consistency between the perpendicular and Hafner methods. Secondary outcome measures were interrater and intrarater agreement of both methods, and normative population data for the distribution of ulnar variance values in children and adolescents for both methods.

### Ulnar variance measurement

Digitalized posteroanterior radiographs of the left hand were previously obtained in all participants [18] and retrospectively used in this study. Radiographs were generated with the shoulder in 90° abduction, the elbow in 90° flexion, and the forearm in neutral rotation, in accordance with recommendations in the literature [19]. Images were standardized into 300 dpi with 12 bits per pixel to facilitate accurate measurements, using a Vidar Diagnostic Pro Advantage scanner using TWAIN v5.2. Images were blinded and skeletal age was determined using automated software (BoneXpert, Visiana) [18].

A musculoskeletal radiologist with 2 years of experience (rater 2) and a fourth-year radiology resident specializing in musculoskeletal radiology (rater 1) measured ulnar variance using step-by-step instructions for both measurements including example images, based on the methods’ descriptions in the literature [11, 12]. To ensure raters’ familiarity with both methods, both raters practiced the use of the Hafner method on 10 images and the perpendicular method on 10 different images that were excluded from further analysis.

In the perpendicular method, the distance from the most distal part of the radius to the adjacent distal cortical rim of the ulna represents ulnar variance (Fig. 1a). The Hafner method [12] consists of two measurements: the distance from the most proximal point of the ulnar metaphysis to the most proximal point of the radial metaphysis (“PRoximal-PRoximal,” or “PRPR”) and the distance from the most distal point of the ulnar metaphysis to the most distal point of the radial metaphysis (“DIstal-DIstal,” or “DIDI”) (Fig. 1b).

### Inter/intrarater agreement and intermethod consistency

Raters independently measured ulnar variance in 60 participants, first using the Hafner method, and then using the perpendicular method on the same images after a 1-week interval, to determine interrater agreement for both methods. In case of a systematic difference between raters for one or both methods, possible causes for discrepancies were discussed during a consensus meeting and measurement instructions were adapted accordingly. Subsequently, raters used the method in question in 60 other participants, and interrater agreement was again determined. This process was set up to be repeated until good interrater agreement, defined by an intraclass correlation coefficient of at least 0.75, was achieved for both methods. For the Hafner method, a single round of 60 measurements was performed to reach this level of agreement, and for the perpendicular method, one consensus meeting and 1-s round of measurements with adapted instructions were carried out.

**Fig. 2:**
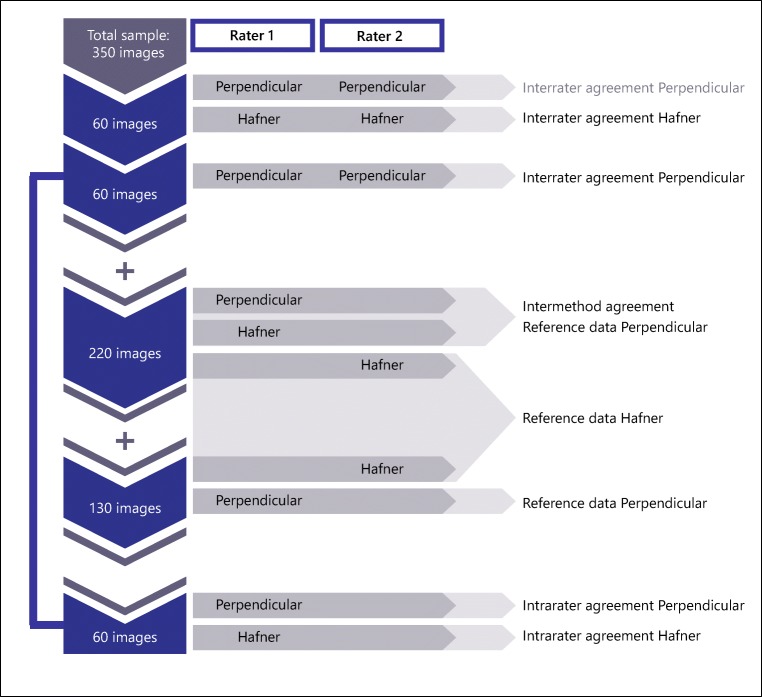
Flowchart showing the inclusion of cases for the various measurements by the two raters

To achieve optimal external validity relating to daily clinical practice, intermethod consistency and intrarater agreement were assessed by the more junior expert (rater 1). For intermethod consistency, rater 1 used both methods to measure ulnar variance in 220 participants in two separate sessions. To determine intrarater agreement for both methods, rater 1 remeasured 60 images in random order and in two sessions (Fig. 2).

### Reference data

In order to also provide reliable population reference data for ulnar variance per skeletal age group, the sample of participants for reliability analysis was augmented until a total of 350 participants was randomly selected from the study population by Van Rijn et al [15]. Rater 1 used the perpendicular method and rater 2 performed the Hafner measurements on all of these images. Reference values for each skeletal age group were calculated for both methods separately.

### Statistical analysis

For assessment of intermethod consistency between the perpendicular and Hafner methods, the intraclass correlation coefficient (ICC) for consistency in rater 1 was calculated using a two-way mixed analysis of variance (ANOVA) model (ICC(3,1)). The average of measurements by the two methods was calculated for each image, as well as the difference in ulnar variance between the two methods. Variability was determined using the method described by Bland and Altman, by calculating the 1.96 standard deviation (SD) of the mean difference between the two methods as the upper and lower limits of agreement [20].

Interrater agreement was assessed in a similar manner: for each method, the ICC for absolute agreement between the raters was calculated using a two-way random ANOVA model (case 2, ICC(2,1)). The means and SD of the measurements were calculated for both raters within each method. The mean difference with its SD between measurements by both raters was calculated, as well as the limits of agreement. From the set of 60 double measurements by rater 1, intrarater agreement for both methods was determined by calculating the ICC for absolute agreement using a two-way random ANOVA model (case 2, ICC(2,2)).

The levels of agreement measured by the ICC were defined as ICC < 0.5 = poor, ICC 0.5–0.75 = moderate, ICC 0.75–0.9 = good, and ICC > 0.9 = excellent. A sample size calculation was done based on an ICC ≥ 0.8 and a preferred 95% confidence interval (CI) of 0.75–0.85, leading to a preferred sample size of at least 201 images to be rated by each rater [21, 22].

## Results

Table 2 shows ulnar variance measurements for the complete cohort and Table 3 for both sexes per skeletal age group.

**Table 2:** Mean ulnar variance for skeletal ages of 5 through 19 years in total cohort

	Perpendicular method			Hafner PRPR			Hafner DIDI		Intermethod ICC_c_ (rater 1)	
Skel. age (years)	No. of cases	Mean ± SD (mm)	Inter rater ICC_a_	No. of cases	Mean ± SD (mm)	Inter-rater ICC_a_	Mean ± SD (mm)	Inter-rater ICC_a_	Perpendicular vs. PRPR	Perpendicular vs. DIDI
5	1	NA	NA	6	− 1.0 ± 1.1	NA	− 2.2 ± 1.4	NA	NA	NA
6	6	− 1.4 ± 0.8	NA	26	− 0.5 ± 1.4	0.91	− 1.5 ± 1.3	0.99	NA	NA
7	13	− 1.9 ± 0.7	NA	23	− 1.0 ± 0.9	0.65	− 1.8 ± 1.1	0.98	NA	NA
8	20	− 1.7 ± 1.0	0.94	22	− 1.6 ± 1.5	0.89	− 2.2 ± 1.3	0.93	0.57	0.73
9	21	− 1.1 ± 0.9	0.92	22	− 1.0 ± 1.3	0.73	− 1.8 ± 1.5	0.93	0.69	0.64
10	24	− 1.4 ± 1.2	0.99	24	− 1.6 ± 2.1	0.99	− 2.2 ± 2.3	0.99	0.64	0.62
11	23	− 1.1 ± 1.1	0.95	23	− 1.0 ± 1.9	0.93	− 2.0 ± 1.6	0.80	0.76	0.66
12	29	− 0.9 ± 1.1	0.86	28	− 0.8 ± 2.1	0.87	− 1.7 ± 1.9	0.95	0.50	0.57
13	35	− 1.5 ± 1.3	0.88	34	− 1.6 ± 1.9	0.86	− 2.5 ± 2.0	0.86	0.69	0.56
14	30	− 1.5 ± 1.6	0.93	25	− 1.4 ± 2.5	0.91	− 2.2 ± 2.1	0.89	0.52	0.64
15	28	− 1.7 ± 1.2	NA	18	− 2.0 ± 1.5	0.44	− 2.9 ± 1.3	0.85	− 0.12	− 0.12
16	35	− 1.3 ± 1.8	NA	17	− 1.3 ± 2.7	0.96	− 2.5 ± 2.7	0.97	0.79	0.82
17	25	− 1.5 ± 1.1	NA	1	NA	NA	NA	NA	NA	NA
18	21	− 1.6 ± 1.7	NA	2	NA	NA	NA	NA	NA	NA
19	1	NA	NA		NA	NA	NA	NA	NA	NA

*Skel. age*, skeletal age; *NA*, not available because of low number of cases; *ICC*_*a*_, intraclass correlation coefficient for absolute agreement; *ICC*_*c*_, intraclass correlation coefficient for consistency

**Table 3:** Mean ulnar variance for skeletal ages of 5 through 19 years for girls and boys

Skel. age (years)	Perpendicular method (mm) Mean ± SD (*n*)		PRPR (mm) Mean ± SD (*n*)		DIDI (mm) Mean ± SD (*n*)	
	Girls	Boys	Girls	Boys	Girls	Boys
5	NA	NA	NA	−1.0 ± 1.5 (4)	NA	− 2.0 ± 1.8 (4)
6	− 1.6 ± 1.2 (3)	− 1.3 ± 0.48 (3)	− 1.2 ± 1.3 (7)	− 0.2 ± 1.4 (19)	− 2.1 ± 1.4 (7)	− 1.2 ± 1.3 (19)
7	− 1.9 ± 0.7 (10)	− 1.8 ± 0.5 (3)	− 1.0 ± 0.9 (12)	− 0.9 ± 0.9 (11)	− 1.7 ± 1.0 (12)	− 1.9 ± 1.2 (11)
8	− 1.7 ± 1.1 (13)	− 1.7 ± 0.9 (7)	− 1.6 ± 1.6 (13)	− 1.4 ± 1.5 (9)	− 2.3 ± 1.3 (13)	− 2.1 ± 1.3 (9)
9	− 0.9 ± 0.8 (14)	− 1.5 ± 1.1 (7)	− 0.9 ± 1.5 (14)	− 1.1 ± 1.2 (8)	− 1.9 ± 1.5 (14)	− 1.8 ± 1.5 (8)
10	− 1.5 ± 1.4 (14)	− 1.1 ± 0.7 (10)	− 2.0 ± 2.5 (14)	− 1.0 ± 1.5 (10)	− 2.7 ± 2.5 (14)	− 1.6 ± 1.8 (10)
11	− 1.0 ± 1.2 (12)	− 1.2 ± 1.4 (11)	− 0.4 ± 2.1 (12)	− 1.7 ± 1.4 (11)	− 1.9 ± 1.4 (12)	− 2.2 ± 1.8 (11)
12	− 0.8 ± 1.4 (13)	− 1.0 ± 0.8 (16)	− 0.5 ± 2.4 (13)	− 1.1 ± 1.8 (16)	− 1.0 ± 2.0 (13)	− 2.2 ± 1.6 (16)
13	− 1.6 ± 1.5 (14)	− 1.4 ± 1.2 (21)	− 1.5 ± 2.0 (14)	− 1.6 ± 1.8 (21)	− 2.3 ± 2.3 (14)	− 2.5 ± 1.7 (21)
14	− 1.8 ± 1.7 (13)	− 1.3 ± 1.6 (17)	− 1.5 ± 2.5 (13)	− 1.7 ± 2.3 (17)	− 2.2 ± 2.6 (13)	− 2.7 ± 1.8 (17)
15	− 1.3 ± 1.6 (12)	− 2.1 ± 0.7 (16)	− 0.7 ± 2.9 (12)	− 2.4 ± 1.1 (15)	− 2.2 ± 2.9 (12)	− 3.1 ± 1.4 (15)
16	− 1.0 ± 1.8 (25)	− 2.1 ± 1.51 (10)	− 0.2 ± 2.6 (21)	− 2.6 ± 1.8 (10)	− 1.2 ± 2.7 (21)	− 3.4 ± 1.9 (10)
17	− 1.4 ± 1.2 (21)	− 1.9 ± 1.0 (4)	− 0.7 ± 1.6 (19)	− 2.1 ± 1.3 (4)	− 1.1 ± 1.7 (19)	− 2.8 ± 1.4 (4)
18	− 1.2 ± 1.2 (13)	− 2.2 ± 2.3 (8)	− 1.0 ± 1.7 (4)	− 1.4 ± 1.1 (5)	− 1.6 ± 1.9 (4)	− 1.5 ± 1.6 (5)
19	NA	NA	NA	NA	NA	NA

*Skel. age*, skeletal age; *NA*, not available because of low number of cases

### Method of perpendiculars

**Fig. 3:**
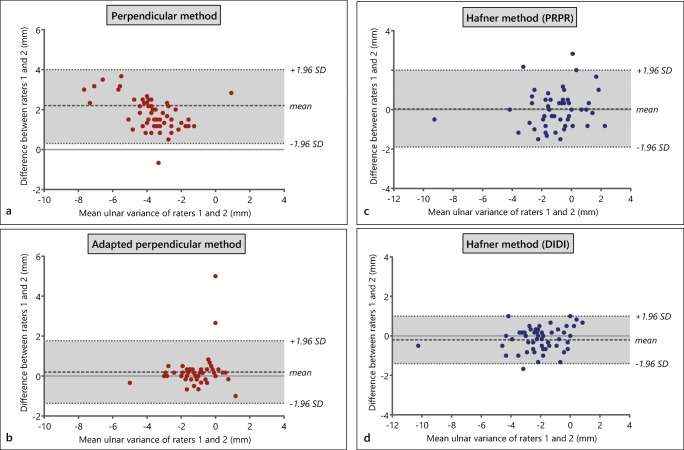
**a** Bland-Altman plot for interrater agreement of the perpendicular method. **b** Bland-Altman plot for interrater agreement of the adapted perpendicular method with extended instructions. **c** Bland-Altman plot for interrater agreement of PRPR of Hafner method. **d** Bland-Altman plot for interrater agreement of DIDI of Hafner method

The ICC for interrater agreement was 0.30 (95% CI, − 0.06 to 0.67) for the perpendicular method, defined as poor. The intrarater ICC was good to excellent: 0.88 (95% CI, 0.70–0.95). The mean systematic difference was 2.2 mm (SD, 0.9 mm) between raters (Fig. 3a), and 0.3 mm (SD, 0.5 mm) within rater 1 (Appendix 1). In 5 cases (8%), the difference between raters was 1 mm or less.

**Fig. 4:**
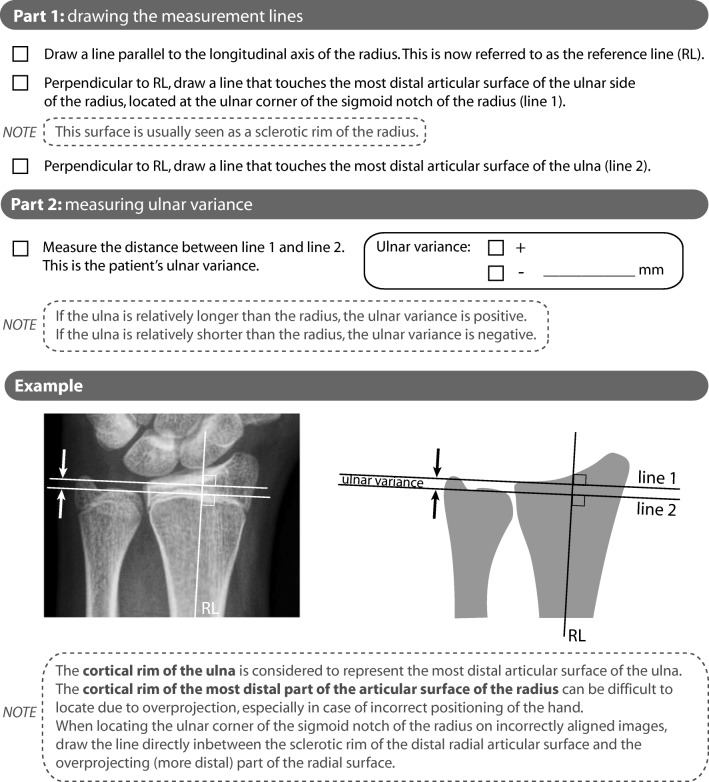
Instructions for the adapted perpendicular method. The complete instruction sheet is provided in Appendix 2

During a consensus meeting, raters concluded that they interpreted the radial surface differently using the literature-based instructions [11]. The perpendicular method’s instructions were adapted into a more detailed description (Fig. 4, Appendix 2) and for the second series of 60 images that were subsequently measured, the mean systematic difference was 0.2 mm (SD, 0.8 mm) and the ICC for absolute agreement of the adapted perpendicular method after one iteration was 0.75 (95% CI, 0.61–0.84), defined as good (Fig. 3b). The complete cohort’s mean ulnar variance was − 1.4 mm (SD, 1.3 mm; range, − 7.0 to 3.5 mm).

### Hafner method

The ICC for interrater agreement for the PRPR distance was good to excellent: 0.88 (95% CI, 0.80–0.93). For the DIDI distance, the interrater ICC was 0.94 (95% CI, 0.90–0.97), defined as excellent. The mean systematic differences were 0.03 mm (SD, 1.0) and − 0.2 mm (SD, 0.6), respectively (Fig. 3).

The intrarater agreement for PRPR was good to excellent as well, 0.90 (95% CI, 0.83–0.94), and for DIDI moderate to good, 0.81 (95% CI, 0.69–0.89), with respective mean systematic differences of 0.02 mm (SD, 0.8) and 0.2 mm (SD, 1.0) (Appendix 1). The difference between raters was 1 mm or less in 42 cases (70%) for PRPR and in 49 (82%) for DIDI. For the complete cohort, the mean PRPR distance was − 1.2 mm (SD, 1.9; range, − 9.5 to 5.3 mm) and the mean DIDI distance was − 2.1 mm (SD 1.8; range, − 10.5 to 3.8 mm).

### Intermethod consistency

**Fig. 5:**
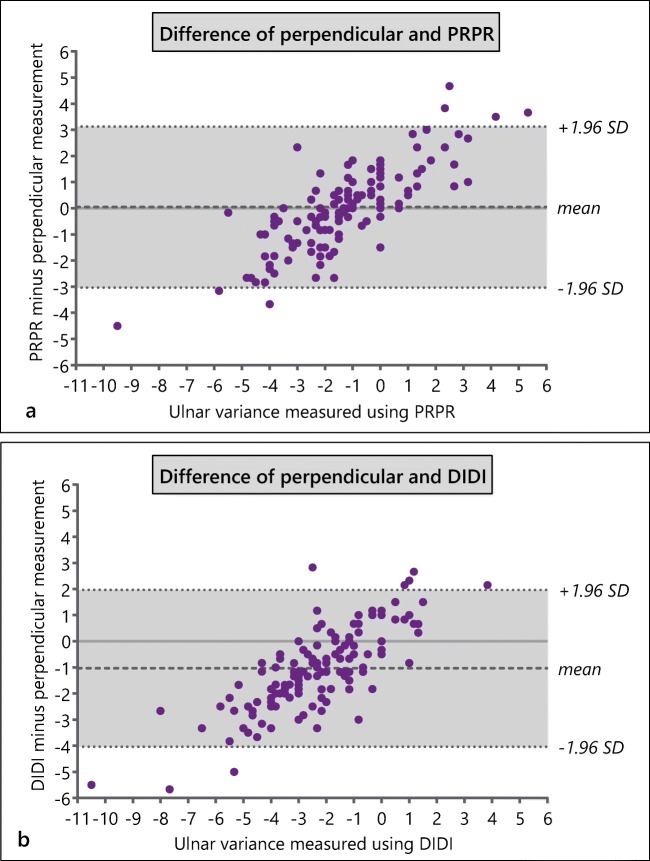
**a** Adapted Bland-Altman plot for Hafner’s PRPR measurement compared with the difference of the PRPR measurement and the perpendicular measurement. In persons in whom both methods can be used, the difference between these measurements can be assessed using these study results. For example: if in a 12-year-old child the PRPR is − 1 mm and the perpendicular method results in an ulnar variance of − 3 mm, the difference between perpendicular compared with PRPR is − 2 mm, which lies within the limits of agreement of the differences found in this study. **b** Adapted Bland-Altman plot for the DIDI measurement compared with the difference of the DIDI measurement and the perpendicular measurement

The ICC for intermethod consistency was 0.60 (95% CI, 0.48–0.70) for the perpendicular method compared with PRPR, defined as moderate. For the perpendicular method compared with DIDI, the ICC for intermethod consistency was moderate as well, with a value of 0.60 (95% CI, 0.49–0.70). Table 2 shows the ICCs for intermethod consistency per skeletal age group. The mean difference between PRPR and the perpendicular measurement was 0 mm, whereas it was − 1 mm between DIDI and the perpendicular measurement (Fig. 5).

**Fig. 6:**
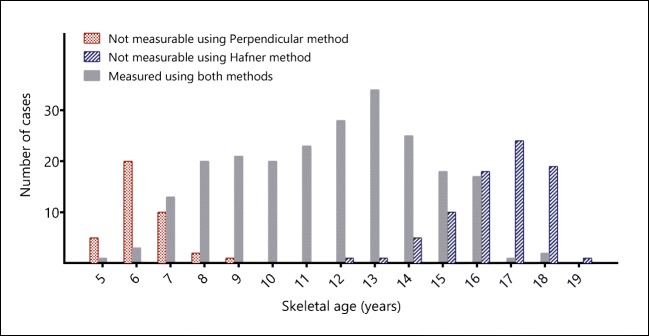
Number of cases in which ulnar variance was measurable with both methods or only with either the perpendicular method or the Hafner method

In 38 cases (11%; 7 girls, 31 boys), all with skeletal ages of 9 years or younger, the perpendicular method could not be used because of absence of the ulnar epiphysis or of both epiphyses (Fig. 6). The Hafner method could not be used in 79 cases (23%; 59 girls and 20 boys), all with a skeletal age of 12 years or older, because one or both growth plates were not visible (Fig. 6).

## Discussion

This study is the first to show moderate intermethod consistency of the perpendicular and Hafner methods for ulnar variance measurement in a population of healthy children and adolescents (ICC 0.60), with reference values for both methods. The interrater agreement was good to excellent for the Hafner method (ICC 0.88–0.94), and good for an adapted version of the perpendicular method with detailed measurement instructions (ICC 0.75) after a consensus meeting.

### Intermethod consistency

In line with previous statements [14], the perpendicular method was moderately consistent with the Hafner method, albeit with dispersed absolute differences between measurements. Figure 5 shows that in negative Hafner measurements, the perpendicular measurement is often more negative, whereas in positive Hafner measurements, the perpendicular measurement is often less positive, and that differences with the perpendicular method are scattered within limits of agreement of − 3 and + 3 mm (PRPR) and − 4 and 2 mm (DIDI). This proportional bias of the perpendicular method compared with the Hafner method likely originates from the different anatomical distances used in these two methods. While PRPR was originally labeled the preferred measurement and is therefore more widely used than DIDI [12], our findings suggest that concomitant use of PRPR and DIDI is valuable for reliable intermethod comparison, but that raters need to take the systematic difference of − 1 mm between the perpendicular method and DIDI taking into account.

### Reliability

The Hafner method’s interrater and intrarater agreement were not reported in the original publication, but one study in young gymnasts illustrated its intrarater reliability with Pearson correlation coefficients of 0.97 to 0.99 [7]. For the perpendicular method, we report an interrater agreement ICC slightly lower than the ICCs of 0.92 (for boys) and 0.89 (for girls) reported earlier [14] that can be (partly) explained by methodological differences. In our study, raters drew all relevant lines while measuring, as opposed to using a template with horizontal lines representing each millimeter of ulnar variance as was done previously [14]. The large discrepancy between inter- and intrarater agreement after the first 60 measurements suggests that even when using the literature-derived instructions, variation between raters can be large. A template might overcome this problem, but may not be available in all PACS systems, warranting clear and unambiguous instructions for those who do not have access to a template. We therefore provide the adapted perpendicular method for ulnar variance measurement use in adults and children with (partly) fused physes and have included a standardized instruction sheet (Appendix 2).

### Reference data

We report comparable pediatric ulnar variance values compared with the commonly used reference values of − 2.1 to − 2.3 mm (PRPR) and − 2.3 to − 2.8 mm (DIDI) reported by Hafner et al, who found 95% confidence interval widths varying from 4 to 9 mm, increasing with age [12]. For the adapted perpendicular method, our results show a more negative ulnar variance than earlier measurements with a slightly lower ICC [14], warranting cautious interpretation. This difference may in part be caused by the adaptation of measurement instructions.

**Fig. 7:**
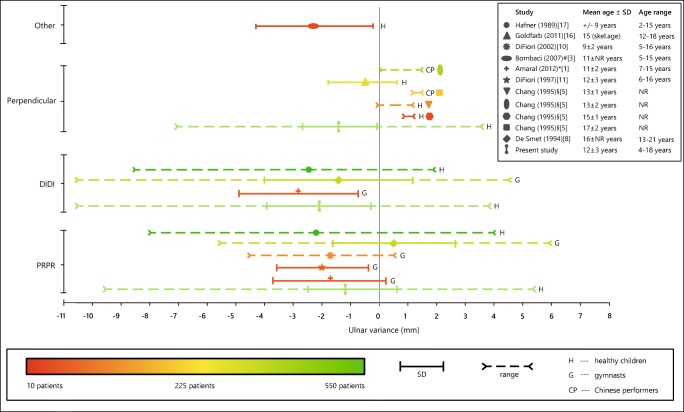
Overview of mean ulnar variance results from the literature. The study’s symbol represents the mean ulnar variance and the sample size is reflected by the symbol’s color. NR, not reported. * Ulnar variance was measured using the Hafner method in immature wrists and the perpendicular method in mature wrists, the average of which is reported. # Ulnar variance was measured on magnetic resonance images. § Mean ulnar variance was not reported

In healthy pediatric populations, mean ulnar variance is reportedly negative: − 2.3 to 0.9 mm (Fig. 7). These studies’ sample sizes and heterogeneity likely have contributed to the large reported confidence intervals compared with the clinically relevant difference of only a few millimeters between negative and positive ulnar variance [7, 12–14, 23–26]. In addition, forearm rotation reportedly affects ulnar variance [19], and although these differences can be small and will therefore not always be clinically relevant [4, 27], slight variations in hand positioning on radiographs may have contributed to the heterogeneity of the population data in the literature. Finally, ulnar variance can increase with age [1, 12, 28], becoming less negative or even positive in adulthood [4]. Our population may have been on average older (chronologically or skeletally) than other pediatric study populations, rendering ulnar variance less negative and closer to adult measurements.

### Strengths and weaknesses

We used radiographs with standardized hand positioning from a large population of healthy children and adolescents without wrist pathology to ensure reliable results and to provide reference data. Although more children aged 12 years and older were included, at least 13 cases per skeletal age group over 6 years were available. Two musculoskeletal radiology specialists first measured several practice cases to prevent bias caused by a learning effect. The methodology included one iteration of adaptation and extension of written instructions for the perpendicular method because of large systematic interrater differences. This resulted in the adapted perpendicular method with improved reliability, which can now be further externally validated in other observers such as hand surgeons or orthopedic surgeons. The reference data represent Western European children and adolescents, and population data need to be established for populations with different ethnicities.

### Clinical impact

Childhood gymnastics performance and distal radial growth plate stress injury are thought to cause increased incidence of positive ulnar variance and long-term consequences like TFCC injury [26, 29]. However, negative, neutral, and positive ulnar variance have all been described in young gymnasts [30], and accurate measurement is therefore essential for future investigations of this relationship. For the diagnosis or therapeutic decision-making process of other conditions related to abnormal ulnar variance, like radial Salter-Harris fractures, Kienböck’s disease, and juvenile idiopathic arthritis [1, 31, 32], reliable measurement of ulnar variance is equally valuable.

The results from this study can aid radiologists, hand surgeons, and other clinicians in choosing the appropriate measurement method and in comparing measurements with reference data, provided from healthy children from Hafner’s age group and from healthy adolescents older than 15 years. For children with skeletal ages of 8 years or younger, the PRPR and DIDI are recommended, and for 14 years or older, the adapted perpendicular method is the measurement of choice. For children with skeletal ages of 9 to 13 years, both methods can be used and measurements can be compared while keeping in mind the − 1 mm systematic difference between the perpendicular and DIDI methods and the higher interrater reliability of the Hafner method. The reference data are organized by skeletal age determined on the same hand radiograph, facilitating maturity-related comparisons.

### Future recommendations

As pediatric mean ulnar variance values vary largely between previous studies, but small changes are suggested to be of influence in various conditions such as wrist pain in gymnasts, future research on the clinically relevant differences in ulnar variance in this population is warranted. Depending on the study population’s age range, the Hafner or perpendicular method should be used to provide accurate measurements. Regardless of the measurement method, standardized wrist positioning should be applied with the forearm in neutral rotation, and caution should be taken that a template may not be useable in all PACS systems and that manual application of measurement lines may be subject to large interrater differences. Use of a standardized instruction sheet (Appendices 2 and 3) can help reduce this variation.

## Electronic supplementary material

ESM 1 (DOCX 8205 kb)

## Abbreviations

DIDI: Distance from the most distal point of the ulnar metaphysis to the most distal point of the radial metaphysis
ICC: Intraclass correlation coefficient
PRPR: Distance from the most proximal point of the ulnar metaphysis to the most proximal point of the radial metaphysis
TFCC: Triangular fibrocartilage complex
