# Supplementary material for: Well-founded practice or personal preference: a comparison of established techniques for measuring ulnar variance in healthy children and adolescents
Source: Eur Radiol. 2019 Aug 7;30(1):151–62. doi: 10.1007/s00330-019-06354-x (PMC6890628; doi:10.1007/s00330-019-06354-x)
Supplement: Supplementary file 1 — (DOCX 8205 kb) [file 330_2019_6354_MOESM1_ESM.docx]

**Electronic Supplementary Material**

**Appendix 1**

**A.** Bland-Altman plot for intrarater agreement of perpendicular method

**B.** Bland-Altman plot for intrarater agreement of PRPR of Hafner method

**C.** Bland-Altman plot for intrarater agreement of DIDI of Hafner method

**Appendix 2.** Ulnar variance scoring sheet for method of perpendiculars

**Appendix 3.** Ulnar variance scoring sheet for Hafner method
